# On the Cause of So-Called Phosphorous Necrosis of the Jaw in Match-Workers

**Published:** 1899-03

**Authors:** Ralph Stockman


### Abstracts and Translations.

### ON THE CAUSE OF SO-CALLED PHOSPHOROUS NECROSIS OF THE JAW IN MATCH-WORKERS.

BY RALPH STOCKMAN, M.D., F.R.C.P.E.[1]

### HISTORICAL SUMMARY.

PHOSPHORUS was first used in the manufacture of matches in 1833, at Vienna. Shortly after its introduction, and ever since, cases of necrosis of the bones of the upper and lower jaws have occurred among the workpeople employed in match-factories. The condition was first described by Lorimer, who, between the years 1839 and 1845, saw nine cases in Vienna, but immediately after cases were also reported in Nuremberg, Strasburg, Berlin, Lyons, Paris, Manchester, and London, as having occurred among the workers in match-factories. Improvements in ventilation and in manufacturing machinery have greatly diminished its frequency, but it has continued to be not uncommon, and is widely recognized as a risk incurred by those who work with phosphorus. The clinical symptoms have been fully described by Lorimer, Heyfelder, von Bibra and Geist, Harrison, Roussel, and others. In addition to their more systematic descriptions, many isolated cases have been put on record by different writers, and all agree substantially in their main features.

### PATHOLOGY.

As regards the bone, the condition is one of cario-necrosis; there is chronic ostitis and periostitis, and it differs in no respect from the same lesion as seen in other bones and from various causes.

[1] Professor of Materia Medica in the University of Glasgow.

It has long been held that this necrosis, or cario-necrosis, as it should rather be termed, is due to a specific action of phosphorous fumes on the bone, these being supposed to cause a peculiar and specific kind of inflammation. If one considers, however, the whole circumstances and the clinical histories of individual cases, the conclusion must inevitably be drawn that the process is due to infection from a micro-organism. Phosphorous fumes consist of phosphorous anhydride ($P_4O_6$) chiefly, with some phosphoric anhydride ($P_2O_5$), and during the oxidation either ozone or hydrogen peroxide is also formed in small amount. From what we know of suppurative processes, it is inconceivable that any of these bodies can cause a chronic purulent inflammation of bone such as has just been described as occurring in "phossy jaw."

Acting on this idea, I applied to Mr. Cornelius E. Garman, surgeon to Messrs. Bryant & May's match-factory, who very kindly has supplied me with specimens of pus from six cases of phosphorous necrosis of the jaw under his care at the present time. In every case the pus was very fetid, and was greenish, or brownish, or grayish in color. Attempts to make cultivations from the pus revealed the presence of staphylococcus albus, streptococci, and numerous other organisms, none of which could reasonably be regarded as the cause of the cario-necrosis.

### PRESENCE OF TUBERCLE BACILLI.

It is well known that tubercle bacilli cannot be cultivated from pus, but on staining cover-glass preparations of the pus by the Ziehl-Neelsen method the bacillus tuberculosis was found in every case. As is usual in the discharge from tuberculous bone, the organisms were few in number and difficult to find except on the closest and most careful examination. On centrifuging the pus and then examining the sediment they were more easily detected. Sometimes several cover-glasses had to be examined before any of the organisms were seen. Most of the bacilli were perfectly typical in appearance, others were small and thick, resembling the forms usually found in the urine. They were scattered about singly, or in small clumps, or in groups of one or several dozens.

Inoculation of guinea-pigs with the pus did not infect these animals with tubercle, and hence the bacilli must be regarded as being either dead or as having almost entirely lost their infective virulence. It is now proved, however, that tubercle bacilli in this condition are quite capable of setting up and maintaining local suppuration and irritation for an indefinite time. Besides, they are assisted by the action of the pyogenic organisms with which the pus swarmed. The condition of the tubercle bacilli is probably to be explained by the fact that all the cases which I have had an opportunity of examining are recovering, and have been under treatment for very long periods with antiseptic mouth-washes, etc. The condition generally is exactly similar to what is seen in tuberculosis of the jaw in cattle, and in tuberculous disease of other bones in man. The presence of the tubercle bacillus can hardly be regarded as fortuitous, seeing that it was found in every case, and its presence is held, so far as our present knowledge goes, at least, to be proof positive of the tuberculous origin of any lesion.

If further proof of the tuberculous nature of the jaw-disease were wanted, it is to be found in looking through the accounts of post-mortem examinations of fatal cases. In most cases death occurs from tuberculosis of the lungs. Whether this is due to infection from the jaw tubercle, or whether the phosphorous fumes damage the lungs, and make them more susceptible to direct infection, I am unable to say.

General tuberculosis is also not uncommon, while tubercle of the abdominal glands and tuberculous ulcers of the intestine are almost invariable, these last arising certainly from infection by swallowing the pus. Abscess in the brain, purulent pleurisy, and tuberculous meningitis are also occasional causes of death. Hectic fever and emaciation always accompany fatal cases.

The part which the phosphorus plays in the process is not far to seek. The acid fumes (phosphorus and phosphoric acids) produced by its oxidation in the air have no effect on bone covered by gum or mucous membrane; but when they can penetrate to the bone directly through the aperture left by a decayed or extracted tooth or any injury, they erode the bone, weaken its nutrition and resisting power at this small spot, and make it susceptible to infection by tubercle bacilli. The bacilli having made good their foothold, spread slowly in some cases and with disastrous rapidity in others. I think I am correct in saying that the great majority of workers in match-factories have carious teeth, and yet only a very small proportion of them become affected with cario-necrosis of the jaw,—namely, those of them who, owing to their home surroundings or to individual predisposition, become readily infected by the tubercle bacillus. Von Bibra and Geist state that the disease may occur weeks or months after the patient has left the match-factory, and in one of their reported cases the woman had actually been eighteen months away from the work before any symptoms began. This in itself is almost complete proof that the phosphorous fumes are only a predisposing cause, and that the disease depends on subsequent infection. It is well known that von Bibra and Geist, and later Wegner, produced suppuration and cario-necrosis in the jaws of rabbits by injuring the periosteum and then exposing the animals to phosphorous fumes (on uninjured rabbits the fumes had no effect). The rabbits all died in from five to ten weeks' time, and were found to have tubercle of the lungs. I experimented in a different way, as it is evident that these animals had become rapidly infected from the laboratory cages in which they were kept. I got new wooden hutches made, placed them in a room where animals had not been previously kept, and kept them scrupulously clean. In the hutches pieces of phosphorus were placed in a mortar on damp earth (to avoid risk of fire) in such quantity that the cages were constantly filled with the fumes in much greater amount than can possibly occur in any factory. Four rabbits were then placed in the hutches after the periosteum and gum had been removed over a considerable portion of the upper and lower jaws in each. In one a tooth was loosened in addition, the operations being all performed under chloroform. They seemed to suffer no inconvenience either from the operation or from living in the phosphorous-fume atmosphere. It has been very difficult to prevent the gum growing over the exposed bone, and after many weeks there is not the slightest trace of any jaw affection. The exposed surface of bone has become slightly eroded and rough, but whether from the action of the acid fumes or from that of the bacilli of the mouth it is impossible to decide.

### TREATMENT.

The treatment hitherto pursued in cases of phosphorous jaw has been to wash out the mouth with deodorant and antiseptic lotions, and wait until the necrosed pieces of bone come away. This is always extremely tedious, and may last many years. In extreme cases the whole lower jaw, or half of it, or parts of the upper jaw have been excised. Sometimes by so doing the whole of the infected portion may be removed, but frequently the disease has again broken out in a neighboring part of the bone. It is evident, however, that early operative interference is called for, and that the original tuberculous focus at the root of the tooth should be removed at once.

PROPHYLAXIS.

As regards prophylaxis, there is absolutely no risk so long as the bone remains protected by gum, and even when carious teeth are present the entrance of the bacilli can be prevented by careful stopping. Efficient ventilation of the workshop will dilute the acid fumes arising from the phosphorus, and make them less active in injuring exposed bone. The infection with the tubercle bacilli is a matter quite apart from the factories, and cannot be controlled either by State regulations or workshop rules. It is acquired—as other tuberculous affections are acquired—by certain persons and not by others, and owing to the present all-pervading frequency of the organism persons with exposed bone eroded by acid fumes, and living under bad hygienic conditions, are very apt to become infected. Whether the fumes also weaken the mucous membrane of the lung alveoli and predispose to pulmonary phthisis among persons employed in match-factories, I have no information which will enable me to decide. It is just possible that actinomyces or other organisms may also occasionally lodge in the weakened bone, and lead to caries and necrosis, but in those cases which I have hitherto examined I have only found the tubercle bacillus.

My great difficulty all along has been to procure a sufficient amount of clinical material to enable me to make my observations more extensive and precise, and I shall be greatly indebted if any surgeon who has cases under his care, and more especially recent ones, will supply me with specimens of the discharge.

In conclusion, I have to express my great indebtedness to Mr. Garman and his son for a great deal of information and assistance, as without their active help and co-operation I could not have made these observations.—*British Medical Journal.*

